# Cytocompatibility of Polymethyl Methacrylate Honeycomb-like Pattern on Perfluorinated Polymer

**DOI:** 10.3390/polym13213663

**Published:** 2021-10-24

**Authors:** Klaudia Hurtuková, Veronika Juřicová, Klára Fajstavrová, Dominik Fajstavr, Nikola Slepičková Kasálková, Silvie Rimpelová, Václav Švorčík, Petr Slepička

**Affiliations:** 1 Department of Solid State Engineering, University of Chemistry and Technology Prague, Technická 3, 166 28 Prague, Czech Republic; klaudia.hurtukova@vscht.cz (K.H.); veronika.juricova@vscht.cz (V.J.); klara.fajstavrova@vscht.cz (K.F.); dominik.fajstavr@vscht.cz (D.F.); nikola.kasalkova@vscht.cz (N.S.K.); vaclav.svorcik@vscht.cz (V.Š.); 2 Department of Biochemistry and Microbiology, University of Chemistry and Technology Prague, Technická 3, 166 28 Prague, Czech Republic; silvie.rimpelova@vscht.cz

**Keywords:** polymer, plasma modification, PMMA, material morphology, honeycomb-like pattern, cell growth, cytocompatibility, FEP, cell adhesion

## Abstract

In this study, we present a simple approach for developing a biocompatible polymer scaffold with a honeycomb-like micropattern. We aimed to combine a plasma treatment of fluorinated ethylene propylene (FEP) substrate with an improved phase separation technique. The plasma exposure served for modification of the polymer surface properties, such as roughness, surface chemistry, and wettability. The treated FEP substrate was applied for the growth of a honeycomb-like pattern from a solution of polymethyl methacrylate (PMMA). The properties of the pattern were strongly dependent on the conditions of plasma exposure of the FEP substrate. The physico-chemical properties of the prepared pattern, such as changes in wettability, aging, morphology, and surface chemistry, were determined. Further, we have examined the cellular response of human osteoblasts (U-2 OS) on the modified substrates. The micropattern prepared with a selected combination of surface activation and amount of PMMA for honeycomb construction showed a positive effect on U-2 OS cell adhesion and proliferation. Samples with higher PMMA content (3 and 4 g) formed more periodic hexagonal structures on the surface compared to its lower amount (1 and 2 g), which led to a significant increase in the pattern cytocompatibility compared to pristine or plasma-treated FEP.

## 1. Introduction

Honeycombs are one of the most studied naturally occurring shapes/structures, which is evidenced by many new reported studies [1]. The impressive hexagonal shape of a honeycomb structure has fascinated many scientists for several decades, especially the research groups of Francois [2,3], Pirk, Zhang [1,4,5,6,7], or Bui et al. [8,9,10]. There were a lot of theories that competed for periodic structures. Two of them have got considerable attention in this field. The first theory states that a bee’s six legs ideally fit onto a hexagon. On the contrary, the second theory supports a mathematical explanation that their geometry enables the coverage of the largest possible area using a minimum of building material [11,12]. However, the latest new theory confirms that hexagonal structures are formed due to highly elastic wax, elevated temperature, and surface tension, transforming circular shapes into hexagonal [12,13,14]. Based on this study, honeycomb-like patterns (HCP) have been identified as unique surfaces due to thousands of interconnected and oriented pores with high mechanical strength [15,16]. Based on the pore size and porosity, three-dimensional (3D) scaffolds resembling the in vivo shape and conditions can be created. This has great potential in biomedical applications, especially in tissue engineering. Porous scaffolds are essential for tissue nutrition, cell proliferation, and the formation of new viable tissues [15,17,18]. Moreover, the porous scaffolds have also protective and storage functions, the latter of which serves for depositing of adhesion molecules and growth factors. Another possible way of utilization is the formation of specific systems for drug delivery [19]. Otherwise, the HCP structures have found a great use in macro-, micro-, and nano-applications in electronics [20], electro-optics [21,22], bioanalytics [23], or in nanofabrication [24].

In several studies, the processes of HCP preparation by various methods including lithography or soft lithography [25,26,27], template techniques [27], emulsions, and plasma etching [28] have been described. However, one of the fundamental techniques invented by Francois et al. has become the so-called breath figure (BF) method. This method has proven to be cost-effective, uncomplicated, flexible, fast, and does not require specific skills to create a micro-/nano-pattern of honeycombs [2,3]. The principle consists of applying a polymer solution in a non-polar solvent to a substrate located in an environment with a relative humidity higher than 50%. A humid atmosphere can be ensured in two ways: static or dynamic. The static method requires atmosphere saturation with water vapor in a closed vessel, while in the dynamic method, the environment is created by the flow of moist air onto the substrate surface [29,30,31]. At the beginning of the process, the solvent is endothermically evaporated from the prepared solution. As the surface temperature decreases, water vapor begins to condense in the form of heterogeneous droplets on the surface of the substrate. After equilibration between the substrate and the environment, the condensed droplets begin to evaporate, thus, spontaneously leaving the hexagonal HCP structures on the substrate surface. The change of key units such as soluble substance, solvent, and humid air creates a wide range of highly ordered porous films with different pore sizes [28,32]. While the BF method requires a humid atmosphere for HCP formation, the newer method of improved phase separation (IPS) invented by Bui et al. [8] requires typical air conditions. The IPS technique uses two steps to prepare thin films on the surface of economically undemanding polymeric materials [8,33,34]. The method is based on a tertiary system: solvent (chloroform)–polymer–”no” solvent (methanol), in the correct volume ratio. To achieve the preparation of HCP structures is necessary to immerse the substrate in the prepared solution and then, spontaneously evaporate it in the air [16,35,36,37]. The advantage of this method, except for the humid environment and other active substances, is the exact ratio of solvents [8].

The development of new methods for creating scaffolds that are structurally and functionally similar to the extracellular matrix is one of the main challenges of tissue engineering. One possibility is to use polymeric materials, mainly due to their simplicity, flexibility, and low cost compared to other materials, and to prove their usefulness as extracellular matrix (ECM) replacements [38]. These properties and many others such as low density, environmental friendliness, and good physical and mechanical properties have been found in polymethyl methacrylate (PMMA) and its derivatives [35], which have been approved as bioinert and biocompatible polymers used in biomedical applications [36,37,39]. Nanocomposites based on PMMA exhibit excellent results in a body environment and have a protective role against corrosion and, thus, increase the durability of biomedical implants [40]. PMMA is commonly used in dentistry in dental prostheses [38,41] as well as bone cement [42,43,44]. Moreover, PMMA has been also used in the field of drug delivery or for the production of contact and implanted lenses, blood pumps and reservoirs, intravenous sets, and blood dialysis membranes [40,45].

In this study, we have focused on the preparation of HCP structures with the addition of PMMA in different amounts/ratios. Surface morphology, chemical composition, wettability, and cytocompatibility of the formed material were investigated for the particular HCP-coated foils. Samples with higher PMMA content formed more periodic hexagonal structures on the FEP surface, as well as cytocompatibility testing of cell culture was more efficient.

## 2. Materials and Methods

### 2.1. Materials and Chemicals

In this study, we used a PMMA polymer foil with a thickness of 50 µm and a density of 1.19 g·cm^−3^, which was purchased from Goodfellow Ltd. (Cambridge, Huntingdon, UK). As solvents, we used methanol (MeOH, HPLC purity, M_r_ = 32.04 g*·*mol^−1^, the density of 0.791 g*·*cm^−3^; purchased from Penta, Prague, The Czech Republic), and chloroform (CHCl_3_, stabilized with ~1% ethanol A.G., M_r_ = 119.38 g*·*mol^−1^, the density of 1.48 g*·*cm*^−^*^3^; purchased from Penta, Prague, The Czech Republic). As a substrate, we used fluorinated ethylene propylene polymer (FEP, the thickness of 50 µm, the density of 2.15 g*·*cm^−3^; purchased from Goodfellow Ltd., Cambridge, Huntingdon, UK).

### 2.2. Pattern Preparation and Modification

The polymer film was treated by Ar^+^ plasma using the SCD 050 sputtering device from BAL-TEC (Baltec AG, Balzers, Liechtenstein). The purity of gas in the chamber was 99.997%, and the pressure was 8 Pa. The samples were placed onto a circular holder (an anode) with a diameter of 10 cm and a distance of 5 cm from the cathode. They were modified at 8 W for 240 s.

In the next step, HCP-like structures were formed using the IPS method via immersion of plasma-modified polymeric substrates in the prepared 100 mL solution for 10 s. The solution was a mixture of two solvents, namely chloroform and methanol, at a volume ratio of 85:15. Then, the amount of 1, 2, 3, and 4 g of PMMA foils was added to the mixture while stirring for 2–3 h at room temperature (22 °C), which gave a homogeneous solution. Then, the HCP-like structures were removed and left to air dry in Petri dishes. After complete evaporation of the solvents, the samples were prepared for further modification and subjected to examination by various analytical methods (see Section 2.3).

### 2.3. Analytical Methods

The wettability of all samples was studied via goniometric measurements of the contact angles, with a drop of distilled water applied to the surface of each sample (6 positions). The contact angles of the samples were determined using a See System goniometer (Advex Instruments, Brno, Czech Republic) at room temperature. A drop of water (8 µL) was applied onto each sample with a Transferpette^®^ automatic pipette (Brand, Wertheim, Germany).

Atomic force microscopy (AFM) was used to study the surface morphology of the FEP substrate with HCP microstructures. The movement of the tip as it passed over the sample was recorded, and a point-by-point image of the surface was compiled. Sample surface morphology was examined by atomic force microscope (AFM) Dimension ICON (Bruker Corp., Billerica, MA, USA); the ScanAsyst mode in the air was used for determination. A silicon tip on a nitride lever SCANASYST-AIR with the spring constant of 0.4 N∙m^−1^ was used. NanoScope Analysis software was applied for data processing. The mean roughness values (R_a_) represent the average of the deviations from the center plane of the sample.

Scanning electron microscopy (SEM) and energy-dispersive X-ray spectroscopy (EDS) were used for detailed analysis of the morphology and chemical characterization of the FEP substrate with HCP microstructures. We used a scanning electron microscope LYRA3 GMU (Tescan, Brno, The Czech Republic) with the accelerating voltage of 10 kV for the electrons that bombarded the samples and an F-MaxN analyzer and SDD (Silicon Drift Detector) (Oxford Instruments, Abingdon, UK) with an applied accelerating voltage of 10 kV for EDS. Platinum (target, purity 99.999%, Safina, Vestec, The Czech Republic) was sputtered onto the samples before analysis using a Quorum Q300T sputtering device (Quorum Technologies, Laughton, East Sussex, England) at a current of 30 mA for 400 s.

### 2.4. Cell Culture

VAMPIRO U-2 OS cells supplied by Innoprot (Derio, Spain) were used to evaluate the material cytocompatibility. The VAMPIRO U-2 OS are cells derived from human osteosarcoma developed through stable transfection of U-2 OS cell line with tFP602 under puromycin resistance gene. The VAMPIRO U-2 OS cells exhibit red fluorescence since they express the red fluorescent protein gene sequence as free cytoplasmatic protein. The cells were cultured in high-glucose Dulbecco’s modified Eagle medium (DMEM; Sigma-Aldrich, St. Louis, MO, USA) containing a stable L-glutamine dipeptide. The medium was supplemented with 10% fetal bovine serum (FBS; certified, Thermo Fisher Scientific, Waltham, MA, USA). The cells were cultivated in a sterile cell culture incubator at 37 °C, atmosphere with 5% CO_2_ and 95% humidity. The cells were regularly passaged when they were at the exponential phase of growth. First, the cells were washed with 5 mL of sterile phosphate-buffered saline (PBS, pH 7.4). After its removal, the cells were detached from the underlying cell culture dish (VWR International, Radnor, PA, USA) with 1 mL of trypsin-EDTA (ethylenediaminetetraacetic acid).

### 2.5. Cell Seeding and Staining

To evaluate the material cytocompatibility, the VAMPIRO U-2 OS cells were used from passages 4–8 (counted from defrosting after the cell delivery). The prepared material samples were subjected to sterilization by immersion into 70% (volume percent) ethanol in water for 30 min. Then, the samples were inserted into cell culture plates with 12 wells (the diameter of 2.14 cm; VWR, Radnor, PA, USA), washed twice with sterile PBS, and weighted down by hollow PMMA cylinders (Zenit, Prague, The Czech Republic). Then, 1 mL of DMEM containing 15,000 cells per one squared centimeter was added to each well onto the evaluated samples in four biological replicates. The endpoints of cultivation were 24 and 96 h, after which the medium was removed, the cells were washed with prewarmed PBS, and fixed with a 4% methanol-free formaldehyde solution in PBS as described in [46]. The fixation occurred for 20 min. at 22 °C in the dark, then, the fixative was removed, and the cells were washed twice with cold PBS. This was followed by staining of the cell structures. Cell nuclei were stained with 0.5 µg·mL^−1^ of 4′,6-diamidino-2-phenylindole (DAPI; Sigma-Aldrich, St. Louis, MO, USA) and cell cytoskeleton was stained with 2 µg·mL^−1^ of phalloidin conjugated to Atto 488 (Sigma-Aldrich, St. Louis, MO, USA) in PBS for 15 min. in the dark at 22 °C. Then, the staining solution was removed, and the cells were washed with cold PBS. The standard deviation was calculated from four biological replicates each including twenty regions of interest, on which the number of grown cells was counted.

### 2.6. Fluorescence Microscopy

The fixed and stained VAMPIRO U-2 OS cell samples were subjected to wide-field fluorescence microscopy using an inverse fluorescence microscope Olympus IX-81 (Olympus, Tokyo, Japan) operated with xCellence software. The fluorescently labeled cells were examined at 100× (10× objective, NA of 0.30), 200× (20× objective, NA of 0.45), and 400× (40× objective, NA of 0.60) magnification. Cell cytoplasm, cytoskeleton, and nuclei were imaged by a triple quad filter DAPI/FITC/TRITC (Olympus, Tokyo, Japan). The images were captured by an EM-CCD camera (Hamamatsu, Honshu, Japan). Then, the fluorescence images were background-corrected, deconvolved (50%), and all three fluorescence channels (DAPI, FITC, TRITC) were merged.

## 3. Results

### 3.1. Surface Morphology Analysis Using SEM and AFM Method

**Figure 1:**
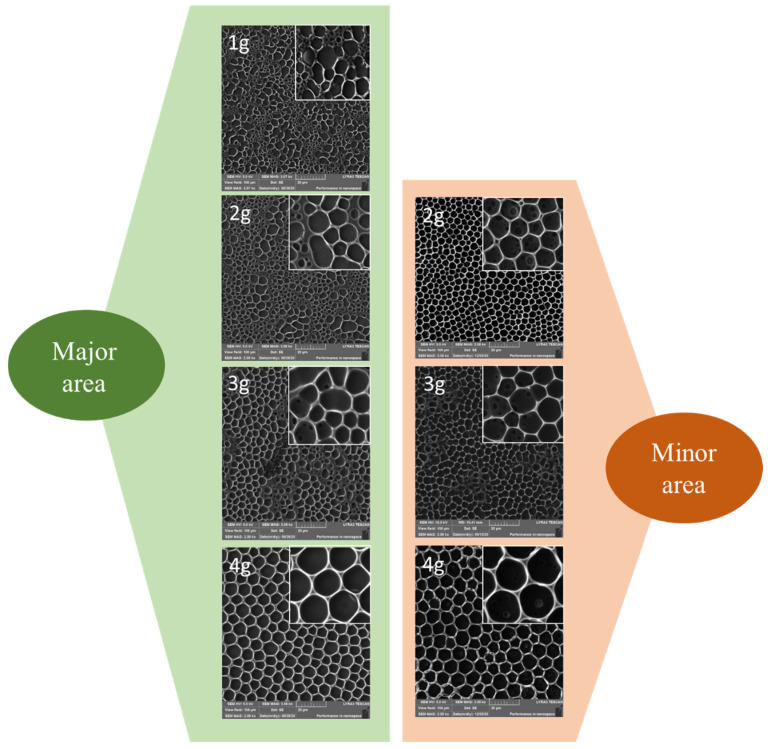
Scanning electron microscopy images of honeycomb-like pattern major and minor areas formed on plasma-modified fluorinated ethylene propylene containing different amounts (1–4 g) of incorporated polymethyl methacrylate. The scanned areas were 100 × 100 µm^2^.

In this study, we have aimed to prepare HCP structures with incorporated PMMA (different content of 1, 2, 3, and 4 g) on plasma-modified FEP matrices. Plasma treatment affects polymer structure resulting in free radical formation. This phenomenon can increase the movement of molecular particles (the ability of a polymer to diffuse) and stimulate the etching rate of the surface layer. The duration of Ar^+^ particle deposition (plasma) significantly affects the polymer topography. Short exposure of a polymer to the plasma treatment creates nanometric structures while changing the polymer surface into a porous form for a longer time [47]. Based on previous research [16,25], the plasma treatment parameters for creating the suitable surface of HCP structures were selected: duration 240 s at the power of 8 W. The changes on the prepared HCP structures’ surface morphology were analyzed by two different methods, AFM and SEM. Figure 1 represents the scans of the prepared samples acquired by the SEM analysis. The analyzed area is divided into two regions for samples with an amount of PMMA higher than 1 g. The first area shows the major heterogeneous site (herein called “major”), which confirms the occurrence of inhomogeneous hexagonal pores. While the second area (herein called “minor”) detected 2 mm from the sample edges of the formed regular hexagonal microstructures. The phenomenon of the split surface is probably caused by different evaporation rates of the solvent mixture on the sample surface, and even though that the difference in patterns was not significant, it was still present on all examined samples, therefore, we wanted to point this out also in the description of Figure 1.

From the SEM images, it can be concluded that a sample containing 1 g of PMMA was the least favorable substrate for forming hexagonal structures. The remaining concentrations of PMMA confirmed the formation of HCP structures. As the amount of PMMA increased, the pore size also increased as well as the pattern homogeneity, and other smaller cavities were layered below each other to different levels. A slight difference in pore sizes was observed when comparing the major and minor areas of HCP on the sample surface. The minor area of the HCP was detected up to 2 mm from the outer diameter. Even the difference in pattern properties in the outer and inner areas was not significant for samples prepared with 3 and 4 g of PMMA, we wanted to introduce this fact in this article for all studied patterns. If we focus specifically on images containing 4 g of PMMA, we conclude that the major area provided pores of up to 4 μm in diameter and the minor area contained also larger pores of up to 10 μm in diameter. In the minor areas, when compared to the major areas, there was a more precise distribution of cavities of almost the same size. These trends were maintained also for samples with a polymer layer containing 2–4 g of PMMA. However, no considerable variation in pore formation was observed for samples formed from a solution containing 3 and 4 g of PMMA.

**Figure 2:**
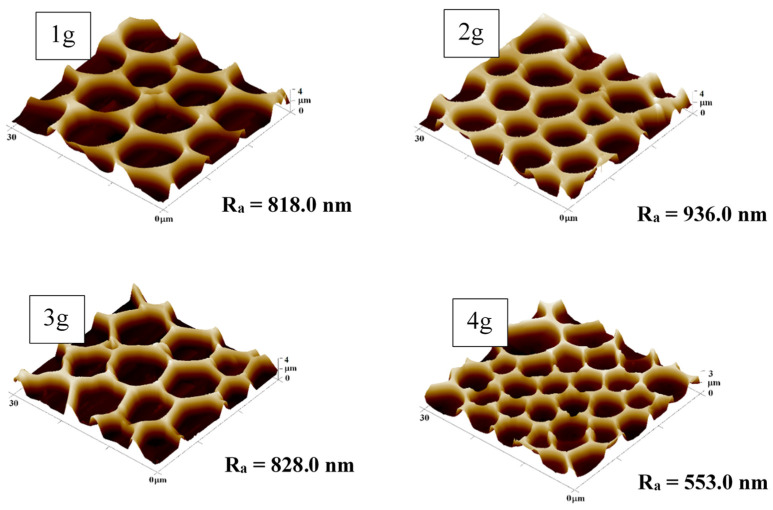
The surface morphology of honeycomb-like pattern minor areas on plasma-modified fluorinated ethylene propylene with different amounts (1–4 g) of incorporated polymethyl methacrylate. The scanned areas were 30 *×* 30 µm^2^. R_a_ represents the average roughness in nm.

Minor areas of the samples with a polymer layer of 1–4 g PMMA were also studied by the AFM analysis as shown in Figure 2. From the results obtained with AFM and SEM, it can be concluded that the pore sizes on the substrates with the same concentration of PMMA remained similar for both studied methods. Therefore, both dimensions and shape of the pattern could be considered as verified. Furthermore, it should be added that the analyzed surface roughness and the height of the pores formed did not change dramatically with the increasing amount of PMMA. The only significant difference among the samples was on the substrate containing the highest content of PMMA (4 g). On the AFM images, we can observe the formation of pores with a slightly smaller diameter in the major area, compared to the edges of the sample (minor area), however, the differences were not significant for samples prepared with 3 and 4 g of PMMA. Even though that the AFM analysis of the sample’s surface was repeated several times. We would like to point out that the SEM scans were acquired on larger sample areas than AFM scans, therefore, the measurement can be considered as more “accurate”.

### 3.2. Surface Chemical Analysis

**Figure 3:**
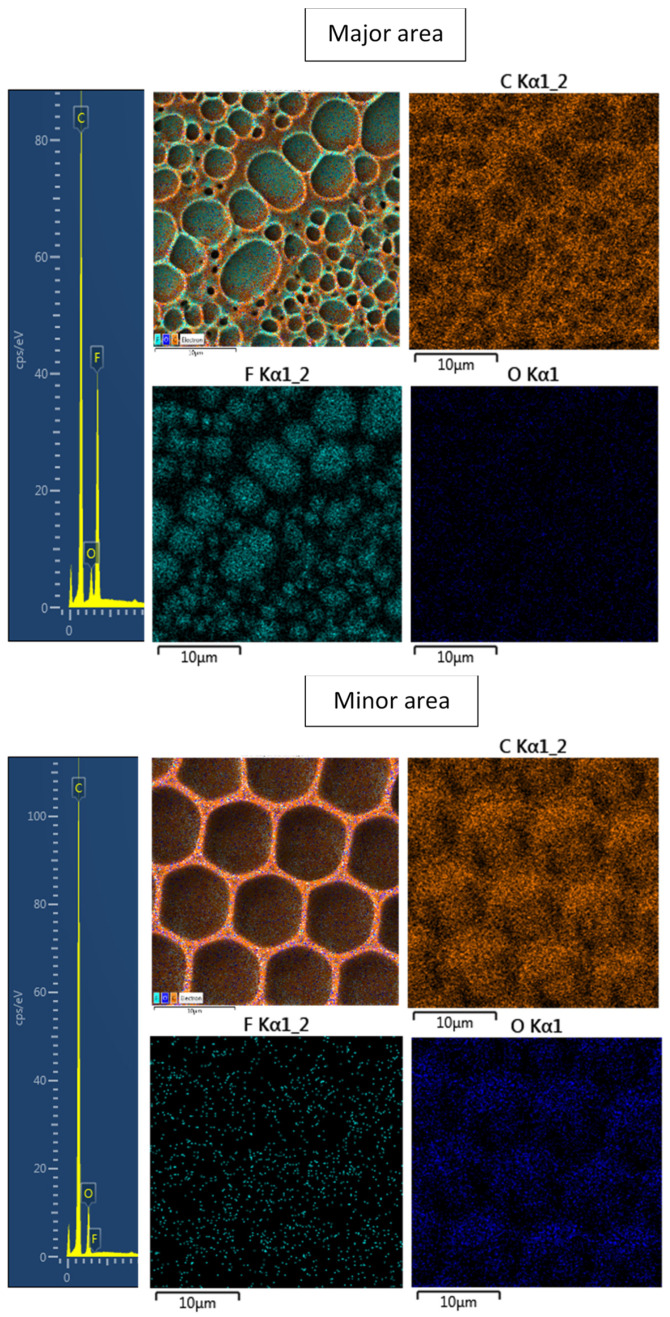
A spectrum from energy-dispersive X-ray spectroscopy (EDS) analysis and elemental maps with layered EDS images for the honeycomb-like pattern major and minor structures on plasma-treated fluorinated ethylene propylene.

Qualitative and quantitative data on the elemental composition of the prepared substrates were determined by the EDS method and are summarized in Table 1 and Figure 3. The samples were analyzed before any modification (pristine FEP), after plasma deposition at 8 W for 240 s (plasma FEP), and after preparation of HCP structures from PMMA solution of various content 1–4 g (HCP + 1 g PMMA). The chemical distribution of major and minor areas of the prepared samples was studied by the EDS technique and described in Section 3.1.

From Table 1, it is evident that no oxygen element was detected on the pristine FEP compared to other modified samples. The explanation is the characteristic of the FEP polymer chain containing only C and F elements [48]. One of the reasons for the increased oxygen concentration is plasma treatment, which causes that the ionized Ar ions to hit the polymer surface. This process induces new functional groups that bind oxygen from the surrounding atmosphere after the sample is removed from the chamber or during the process if a slight amount of oxygen is present in the atmosphere in the chamber [49,50]. Another reason is the presence of PMMA honeycombs, which contain a large amount of oxygen in their structure [51]. The subsequent phase separation increases the carbon content and, conversely, decreases the fluorine concentration, confirming a polymeric PMMA layer formation. The presence of PMMA may limit the content of the F element, as shown in Table 1. The highest tested amount of PMMA (4 g) formed surface with minimum fluorine content, which was only 6.4%. It is interesting to note that if we observed a minor area of the sample (minor), the F element almost disappeared, i.e., the substrate was fully covered with a PMMA polymer layer. For a better interpretation of the surface’s chemical composition, the scans from EDS of the sample with 2 g PMMA are attached in Figure 3.

### 3.3. Surface Wettability

Wettability, i.e., the contact angle values, is a crucial property for material characterization. It plays an essential role in applications of technological (cleaning, gluing, dyeing) [52,53] or medical processes (cell or bacterial adhesion) [54]. The value of a contact angle has a significant impact on the material surface and characterizes the hydrophilicity/hydrophobicity of a polymer [55].

**Figure 4:**
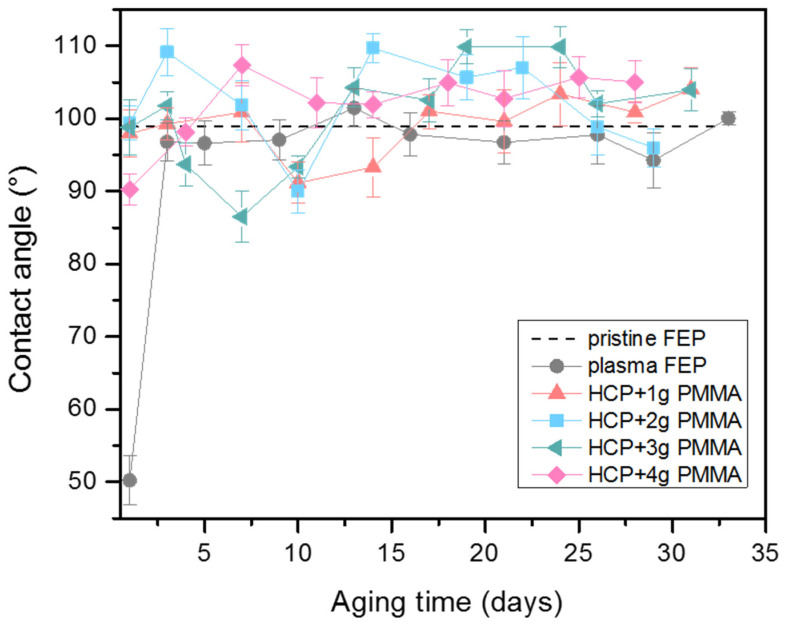
Water contact angles measurements over 34 days for pristine fluorinated ethylene propylene (FEP), plasma-treated FEP (plasma FEP), and honeycomb-like pattern (HCP) structure with incorporated polymethyl methacrylate (PMMA) of various content (1–4 g).

The graph shown in Figure 4 provides data on the surface wettability of all evaluated samples during an aging period of 34 days. The aging study was performed on the unmodified FEP substrate (pristine FEP), plasma-modified FEP substrate at 8 W for 240 s (plasma FEP), and the substrate after plasma modification applied with a layer of PMMA at various amounts (1–4 g). The value of the contact angle of the unmodified FEP sample was 98.9°. As it is obvious from Figure 4, the samples with HCP had contact angles in the region of slight hydrophobicity ranging from 90–110° with fluctuation during the aging period. However, the slightly hydrophobic character remained unchanged and stable during the aging period. The most apparent change in contact angle values was detected for the plasma-modified FEP substrate. On the first day, the plasma FEP had a contact angle of 50.2°, and on the third day, it increased rapidly to 96.8°. Contact angle did not change significantly throughout the measurement and was around the values of the pristine FEP. This could be caused by the redirection of polar groups from the material’s surface to its volume due to the preservation of samples in the ambient atmosphere. On the other hand, an atmosphere with relatively low humidity and temperature can slow down the material aging process. All FEP samples covered with PMMA maintained a hydrophobic surface with contact angles within 10° of the value of unmodified FEP (98.9°). The influencing of contact angle by various patterns including honeycomb structure was reported in [56], where also superhydrophibic effect was observed.

### 3.4. Cytocompatibility

**Figure 5:**
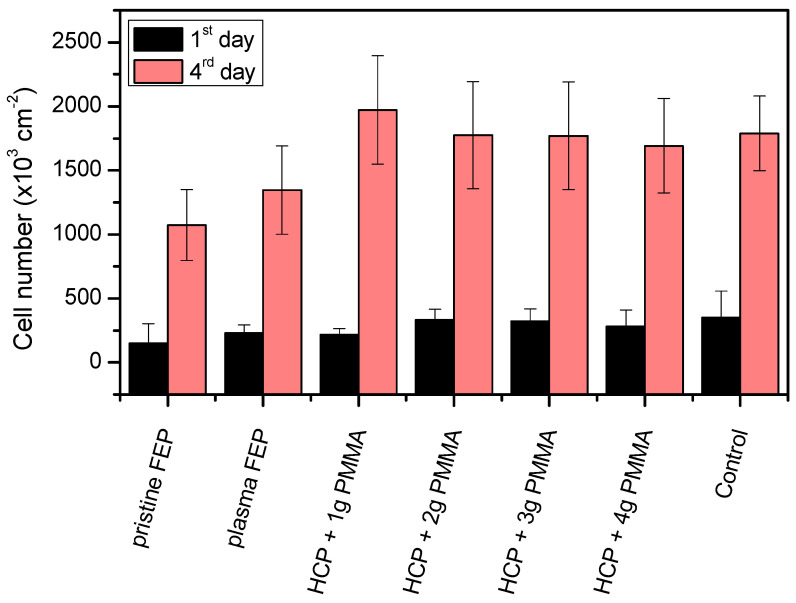
The number of adhered (24 h) and proliferated (96 h) VAMPIRO U-2 OS cells on a pristine and plasma-treated (plasma) fluorinated ethylene propylene (FEP) film and plasma-treated FEP films subsequently coated with honeycomb-like patterns formed from different polymethyl methacrylate (PMMA) solutions (1–4 g) and tissue culture polystyrene (TCPS, control).

Human cells derived from osteosarcoma stably transfected with a red fluorescent protein tFP602 (VAMPIRO U-2 OS cells) were chosen as a model cell line for cytocompatibility measurement of the prepared materials. Samples of pristine FEP, plasma-modified FEP, and FEP with a PMMA layer were subjected to this study. Tissue culture polystyrene (TCPS) was chosen as a control since this material is routinely used for tissue culture. From the plot in Figure 5, it is evident that after 24 h post-seeding, there was no statistically significant difference in the number of cells adhered on modified or unmodified samples compared to the control sample of TCPS. It can be caused by the cell adaptation process to the samples after their seeding [57]. After additional cultivation, 96 h post-seeding, cell proliferation was obvious on each evaluated sample. The lowest number of cells was detected on the unmodified FEP sample, which is consistent with our previous results, e.g., [58,59,60]. The cell number was increased on plasma-treated FEP 96 h after seeding when compared to pristine FEP. This was caused by the plasma exposure, which creates new oxygen-containing groups (carbonyl and carboxyl mainly) in combination with significant wettability change, enabling the design of excellent scaffolds with increased biocompatibility [60]. However, the best cytocompatibility was observed on FEP samples with HCP structures and PMMA layers, since a markedly pronounced increase in the cell number was detected. The number of cells of these samples was equal to that of the control TCPS sample. The improved VAMPIRO U-2 OS cell proliferation on the modified samples might be caused by the porous character of the HCP-modified sample surface or the presence of PMMA. The presence of 3D HCP structure mimics the extracellular matrix necessary as scaffolds for proper cell growth and proliferation [61], the effect also observed for laser induced periodic surface structures [24,62]. On the other hand, the PMMA material exhibits biocompatible properties, such as surface chemistry in combination with morphology properties [36,37,39,40] that could increase cell proliferation on the substrate surface compared to pristine and plasma-modified FEP.

**Figure 6:**
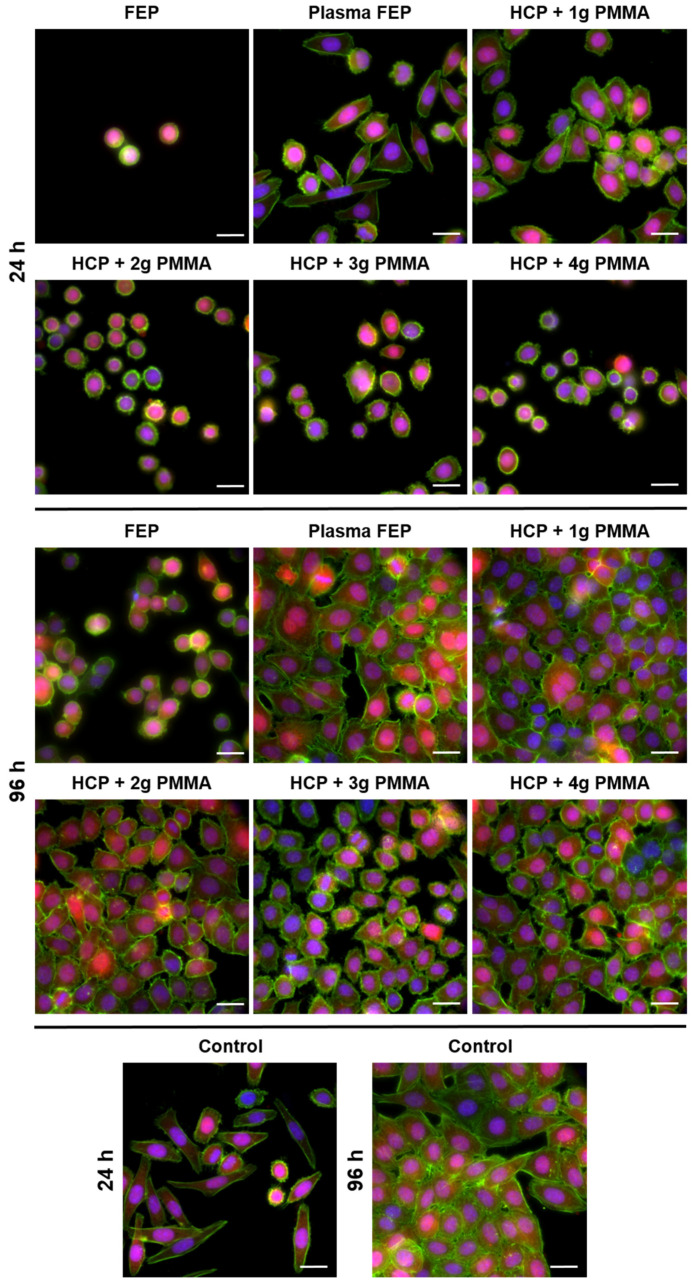
Fluorescence microscopy images of VAMPIRO U-2 OS cells (human cells derived from osteosarcoma, stably transfected with tFP602) growing on fluorinated ethylene propylene (FEP) treated by 8W plasma for 240 s and subsequently coated with a honeycomb-like pattern formed from polymethyl methacrylate (PMMA) solution containing its different amount (1, 2, 3, and 4 g). Left and right columns-cells 24 and 96 h post-seeding, respectively. As controls, tissue culture polystyrene (TCPS), pristine FEP, and plasma-treated (8 W, 240 s) FEP matrices were used. In green––cell cytoskeleton, in blue––cell nuclei, in red––cell cytoplasm. Scale bar represents 20 μm.

Another evaluation of the cellular response, morphology, was studied on samples with cultured U-2 OS cells for 1 and 4 days, shown in Figure 6. Images of the cell samples were captured by fluorescence microscopy. After the first day, round cells were detected with irregular spreading on pristine FEP samples and with a layer of 3 g and 4 g of PMMA, which did not correspond to the shape of osteoblasts. In comparison, the control sample showed a cell morphology similar to the shape of osteoblasts. Samples with plasma modification and 1 g and 2 g PMMA had a cell shape similar to the control, but their distribution was not regular. After day 4, all prepared samples were completely covered with cells, except for the pristine FEP sample. This phenomenon could be due to the smooth surface of the unmodified FEP. The shape of the cells after day 4 can no longer be recognized in all sample types.

## 4. Conclusions

In this study, we prepared an HCP from PMMA on plasma-treated perfluorinated polymer FEP. The plasma treatment was confirmed to play a crucial role in the pattern formation since the surface wettability and chemistry were significantly altered and, thus, the process of IPS was successfully applied. The pattern aging revealed the stabilization of contact angle values in a range of 90–110° for all types of studied PMMA micropatterns. The surface morphology of the HCP was confirmed by both AFM and SEM, by which we revealed the formation of two areas of PMMA micropatterns on the FEP surface. The micropatterns slightly varied in their surface properties, which was verified by SEM surface mapping. Pristine unmodified FEP did not support the adhesion and proliferation of human osteoblasts (VAMPIRO U-2 OS cells) due to low surface wettability, roughness, and the absence of oxygen. Further, we have confirmed that the plasma exposure of FEP itself slightly improved the cytocompatibility of this substrate. The subsequent process of HCP formation from PMMA led to improvement of adhesion and growth of VAMPIRO U-2 OS cells, and the cells were able to grow in clusters according to the shape of the pattern even after 96 h of cultivation. This study also opens the wide possibility to prepare surfaces with the possibility of drug release due to an extreme increase in effective surface area for particular HCP structures.

## Figures and Tables

**Table 1:** The atomic concentrations of fluorine, oxygen, and carbon in at. % were determined using the energy-dispersive X-ray spectroscopy for unmodified pristine fluorinated ethylene propylene (FEP), plasma-treated FEP, and with a honeycomb-like pattern (HCP) major and minor structure with incorporated polymethyl methacrylate (PMMA) of various content (1–4 g).

Sample	C [%]	O [%]	F [%]
Pristine FEP	45.5	0.0	54.5
Plasma-treated FEP	41.9	1.8	56.3
HCP + 1 g PMMA (major)	58.3	2.8	38.9
HCP + 2 g PMMA (major)	74.0	3.6	22.4
HCP + 3 g PMMA (major)	74.3	4.1	21.6
HCP + 4 g PMMA (major)	86.4	7.2	6.4
HCP + 2 g PMMA (minor)	93.6	6.3	0.1
HCP + 3 g PMMA (minor)	91.0	6.6	2.4
HCP + 4 g PMMA (minor)	92.9	7.0	0.1

## Data Availability

Not applicable.

## Abbreviations

3D	Three dimensional
AFM	Atomic force microscopy
BF	Breath figure
EDS	Energy-dispersive X-ray spectroscopy
FEP	Fluorinated ethylene propylene
HCP	Honeycomb-like pattern
HPLC	High-performance liquid chromatography
IPS	Improved phase separation
PMMA	Polymethyl methacrylate
SEM	Scanning electron microscopy
TCPS	Tissue culture polystyrene
U-2 OS	Human cells from osteosarcoma
